# Tumeurs cérébrales de l’enfant: à propos de 136 cas

**DOI:** 10.11604/pamj.2018.30.291.13208

**Published:** 2018-08-24

**Authors:** Fatima-Ezzahra Hazmiri, Fatima Boukis, Said Ait Benali, Najat Cherif Idrissi El Ganouni, Hanane Rais

**Affiliations:** 1 Service d’Anatomie Pathologique, CHU Mohammed VI, Hôpital Arrazi, Marrakech, Maroc; 2 Service de Neurochirurgie, CHU Mohammed VI, Hôpital Arrazi, Marrakech, Maroc; 3 Service de Radiologie, CHU Mohammed VI, Hôpital Arrazi, Marrakech, Maroc

**Keywords:** Tumeurs cérébrales, enfant, épidémiologie, histopathologie, Brain tumors, child, epidemiology, histopathology

## Abstract

Les tumeurs cérébrales sont les tumeurs solides les plus diagnostiquées chez les enfants de moins de 15 ans dans le monde. Toutefois, peu de publications ont rapporté le profil épidémiologique et anatomopathologique de ces tumeurs en Afrique et particulièrement au Maroc. Les auteurs rapportent les particularités épidémiologiques et anatomopathologiques des tumeurs cérébrales primitives de l'enfant dans la région de Marrakech (Sud du Maroc). C'est une étude rétrospective réalisée au service d'anatomie pathologique du CHU Mohammed VI de Marrakech de 2004 à 2016. Cent trente-six cas de tumeurs cérébrales primitives étaient diagnostiqués avec une moyenne de 11,33 cas par an. L'âge moyen était de 8,28 ans. Le sex-ratio (H/F) était de 1,6 avec une légère prédominance masculine. Ces tumeurs étaient infra-tentorielles dans 61,53% des cas et siégeaient en supra-tentoriel dans 38,47% des cas. A l'étage infra-tentoriel, les tumeurs des hémisphères cérebelleux occupaient le premier rang (61,4%). Parmi les dix-huit types histologiques diagnostiqués, l'astrocytome et le médulloblastome représentaient ensemble 46,32% (29,41% et 16,91% respectivement). Dans notre contexte, la majorité des tumeurs cérébrales de l'enfant prédominait dans les 2 groupes d'âge: 5-9 ans et 10-15 ans. Les résultats épidémiologiques de ces tumeurs au sud du Maroc concordent majoritairement avec ceux déjà publiés du Nord du pays et des autres pays non Africains.

## Abstract (en)

Brain tumors are the most diagnosed solid tumors in children under the age of 15 years worldwide. However, the epidemiological and anatomopathological profile of these tumors has been poorly described in African and, particularly, in Moroccan literature. This study highlights the epidemiological and anatomopathological peculiarities of primary brain tumors in children living in the region of Marrakech (south Morocco). We conducted a retrospective study in the Division of Anatomic Pathology at the Mohammed VI University Hospital, Marrakech from 2004 to 2016. One hundred and thirty-six patients were diagnosed with primary brain tumor, a mean of 11.33 cases per year. The average age of patients was 8.28 years. Sex-ratio (M/F) was 1.6 with a slight male predominance. Infratentorial tumors were found in 61,53% of cases while supratentorial tumors were found in 38.47% of cases. Infratentorial tumors mainly occurred in the cerebellar hemisphere (61.4%). Eighteen histological types were diagnosed. Astrocytoma and medulloblastoma accounted for 46,32% (29.41% and 16.91%, respectively). In our context, the majority of brain tumors in children was predominant in both age groups: 5-9 years and 10-15 years. The epidemiological data of these tumors from south Morocco are mostly consistent with those already published in North Country’s literature and in other non-african countries’ literature.

## Introduction

Les tumeurs cérébrales de l'enfant (TCE) sont les tumeurs solides pédiatriques les plus fréquentes. Elles représentent 20% des affections malignes de l'enfant et constituent la deuxième cause de décès par cancer après les leucémies dans cette classe d'âge [1-3]. Plusieurs aspects les différencient des tumeurs cérébrales de l'adulte, notamment leurs types histologiques et leur topographie [2,4]. Certes, quelques séries marocaines ont dressé le profil démographique de ces tumeurs, toutefois, leurs résultats restent restreints au nord du pays [5,6]. Le but donc de ce travail est d'évaluer à travers une série de 136 cas, les particularités épidémiologiques et anatomopathologiques de ces tumeurs dans la région de Marrakech au Sud du Maroc.

## Méthodes

Il s'agit d'une étude rétrospective de 136 cas de tumeurs cérébrales pédiatriques primitives diagnostiquées au laboratoire d'Anatomie Pathologique, au CHU Mohammed VI de Marrakech sur une période de 12 ans allant de Janvier 2004 à Décembre 2016. Le diagnostic histopathologique a été porté sur des prélèvements biopsiques ou des pièces d'exérèse opératoires d'enfants âgés de 0 à 15 ans. Ces prélèvements ont été examinés après fixation au formol à 10%, inclusion en paraffine, coupés à 4 µ et colorés par coloration standard hématoxyline-éosine (HE). La lecture était effectuée au microscope optique aux différents grossissements. Les lésions métastatiques, kystiques et malformations vasculaires ont été exclues. Les types histologiques ont été déterminés selon la classification de l'Organisation Mondiale de la Santé (OMS). Cette analyse a porté sur les caractéristiques épidémiologiques et anatomopathologiques des enfants atteints de tumeurs cérébrales primitives dans notre région.

## Résultats

**Figure 1:**
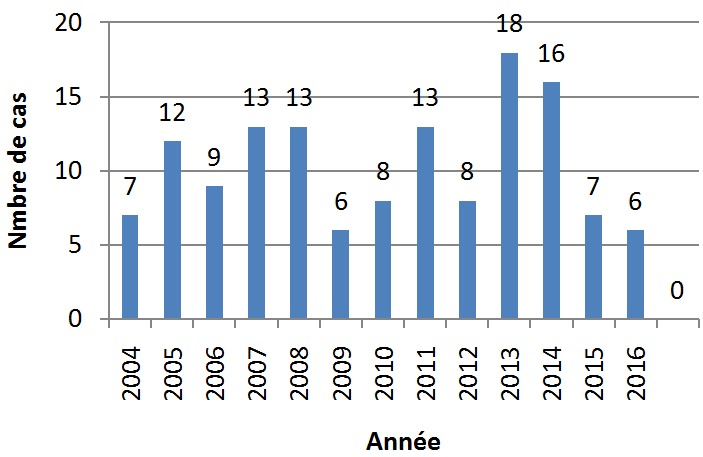
Répartition des cas selon les années

**Figure 2:**
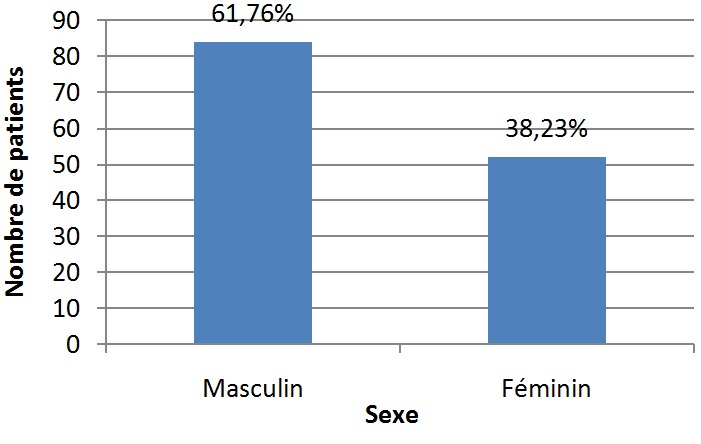
Répartition des patients selon le sexe

**Figure 3:**
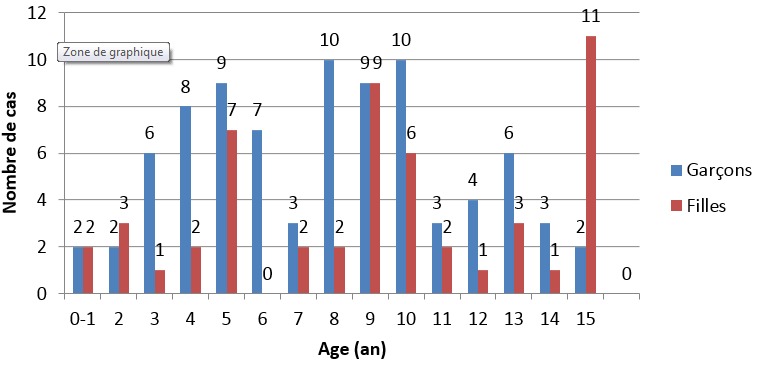
Répartition des tumeurs selon l’âge et le sexe

**Figure 4:**
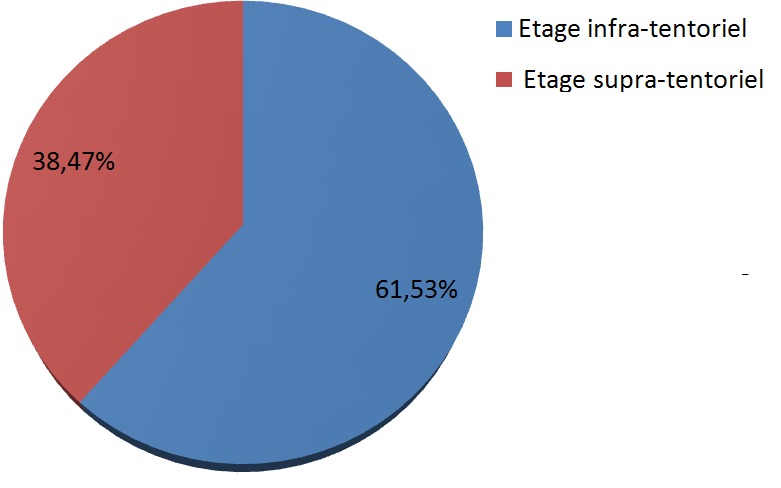
Répartition des tumeurs selon leur topographie

**Figure 5:**
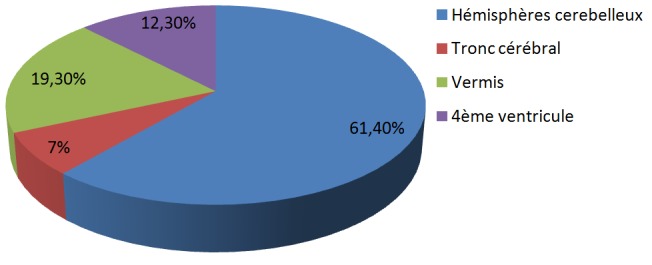
Topographie des tumeurs infra-tentorielles

**Figure 6:**
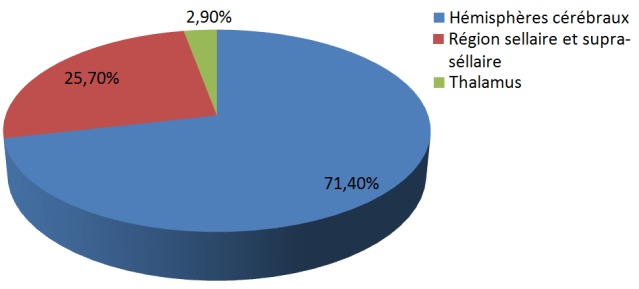
Topographie des tumeurs supra-tentorielles

**Figure 7:**
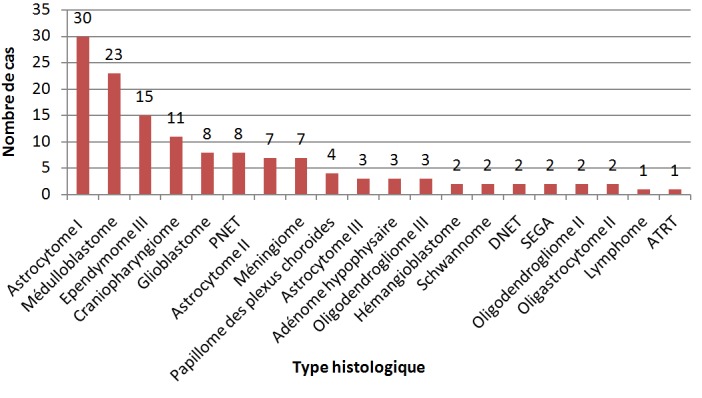
Répartition des tumeurs selon le type histologique

Cent trente-six cas de tumeurs cérébrales primitives ont été diagnostiqués avec une moyenne de 11,33 cas par an (Figure 1). Une légère prédominance masculine a été observée (61,76% de sexe masculin contre 38.23% de sexe féminin) avec un sex-ratio (H/F) de 1,6 (Figure 2). L'âge moyen était de 8,28 ans (0-15 ans). Autant de garçons que de filles étaient affectés dans la tranche d'âge 0-1an et à l'âge de 9 ans. Les TCE étaient beaucoup plus diagnostiquées chez les filles que les garçons à l'âge de 15 ans (84,61% de filles contre 15,38% de garçons). Dans les tranches d'âge 2-8 ans et 10-14 ans, ces tumeurs étaient plus rencontrées chez les garçons que les filles (62,22% et 52% de garçons contre 37,77% et 48% de filles respectivement) (Figure 3). Sur le plan topographique, 61,53% des tumeurs siégeaient au niveau de l'étage infra-tentoriel et 38,47% à l'étage supra-tentoriel (Figure 4). Les tumeurs infra-tentorielles étaient dominées par les tumeurs des hémisphères cérébelleux (61,4%) (Figure 5) et les tumeurs supra-tentorielles étaient dominées par les tumeurs des hémisphères cérébraux (71,40%) (Figure 6). Dix-huit types histologiques étaient diagnostiqués, dont deux prédominaient à savoir les astrocytomes I, II et III (29,41%) et les médulloblastomes (16,91%) (46,32%; près de la moitié des cas ensemble). Les astrocytomes pilocytiques (grade I) à eux seuls représentaient 22% de toutes les tumeurs. Ces astrocytomes -tout grade confondu- étaient suivis des épendymômes III (11%), craniopharyngiomes (8%), glioblastomes (5,88%), tumeurs neuro-ectodermiques primitives (PNET) (5,88%), méningiomes (5,14%), papillomes des plexus choroides (2,94%), oligodendrogliomes III (2,2%)et adénomes hypophysaires (2,2%). Les tumeurs neuro-épithéliales dysembryoblastiques (DNET), les astrocytomes sus- épendymaires à cellules géantes (SEGA),les schwannomes, les oligodendrogliomes II, les oligoqstrocytomes II, hémangioblastomes et la tumeur rhabdoide tératoide atypique (ATRT) représentaient 10,44% (Figure 7).

La majorité des tumeurs dans notre contexte prédominait dans les 2 groupes d'âge: 5-9 ans et 10-15 ans (80,88%) (Tableau 1): 1) l'astrocytome I: 96,66%; 2) le médulloblastome: 86,95%; 3) le craniopharyngiome: 90,9%; 4) le méningiome: 85,71%; 5) la PNET: 75%; 6) l'astrocytome II: 71,42%; 7) l'astrocytome III: 66,66%; 8) l'oligodendrogliome III: 66,66%; 9) l'épendymome III: 60%; le glioblastome: 62,5%. Le schwannome, l'adénome hypophysaire, la DNET, la SEGA, l'hémangioblastome, l'oligodendrogliome II et l'oligoastrocytome II étaient exclusivement diagnostiqués dans ces 2 groupes d'âge: 100%. Le papillome des plexus choroides était plus retrouvé chez les enfants des 2 groupes 0-2 et 3-4 ans: 75%. Les 2 cas; lymphome et ATRT étaient exclusivement notés dans le groupe 3-4 ans.

**Tableau 1:** Répartition des types histologiques selon les groupes d’âge

	Nombre de cas/groupes d’âge			
Type histologique	0- 2ans	3- 4ans	5 - 9ans	10 -15ans
Astrocytome I	-	1	13	16
Médulloblastome	-	3	15	5
Ependymome III	2	4	6	3
Craniopharyngiome	1	-	5	5
Glioblastome	1	2	2	3
PNET	-	2	3	3
Astrocytome II	2	-	3	2
Méningiome	-	1	3	3
Papillome des plexus choroides	2	1	1	-
Astrocytome III	1	-	1	1
Adénome hypophysaire	-	-	1	2
Oligodendrogliome III	-	1	1	1
Hémangioblastome	-	-	-	2
Schwannome	-	-	1	1
DNET	-	-	-	2
SEGA	-	-	1	1
Oligodendrogliome II	-	-	1	1
Oligastrocytome II	-	-	1	1
Lymphome	-	1	-	-
ATRT	-	1	-	-

## Discussion

Les tumeurs cérébrales sont la deuxième cause de cancer chez l'enfant. Les tumeurs primitives prédominent et les types histologiques sont très variés [1,2]. Toutefois, les particularités démographiques de ces tumeurs sont peu rapportées dans la littérature notamment en Afrique [5] et particulièrement au Sud du Maroc. Les résultats de notre étude menée dans la région de Marrakech (CHU Mohammed VI) concordent majoritairement avec ceux déjà publiés du nord du pays [5,6] et des autres pays Africains et non Africains [7,8]. Le nombre de cas par an dans notre étude (11,33) était inférieur à celui constaté dans la littérature en général [9-11] et particulièrement à celui rapporté au Nord du pays [6]. En effet, notre institution compte parmi les centres hospitalo-universitaires assez jeunes au Maroc. La répartition selon le sexe est généralement égale [2]. Quelques séries ont rapporté une prédominance masculine [3,4,8,12]. Nos résultats où 61,76% des cas étaient des garçons concordent globalement avec ces données. Le sexe ratio H/F noté dans notre étude (1,6) était légèrement supérieur à celui retrouvé au Nord du pays [5,6]. L'âge moyen rapporté dans notre série (8,28 ans) est proche de celui retrouvé dans les séries marocaines [5,6] et une série Algérienne [7].

Le diagnostic topographique retrouvait la localisation infra-tentorielle dans plus de la moitié des cas (61,53%), particulièrement au niveau cérébelleux, ce qui est concordant avec les données de la littérature [4-6]. La classification anatomo-pathologique de l'OMS, actualisée en 2016, est utilisée pour classer les tumeurs du système nerveux central [13]. Les astrocytomes pilocytiques, les médulloblastomes, les autres gliomes et les épendymomes représentent plus de 80% des tumeurs de l'enfant [6,14]. Le glioblastome est 100 fois moins fréquent que chez l'adulte. La fréquence des différents types histologiques dépend de l'âge [2,15]. Les résultats concernant le type histologique dans notre étude sont similaires à ceux de la littérature. En effet, parmi les 18 types histologiques diagnostiqués, les astrocytomes (29,41%) et les médulloblastomes (16,91%) représentaient ensemble près de la moitié des cas). Les astrocytomes pilocytiques (grade I) à eux seuls représentaient 22% de toutes les tumeurs. Les épendymômes III étaient au 3^ème^ rang (11%) et les craniopharyngiomes au 4ème rang (8%). La majorité de ces tumeurs est diagnostiquée après l'âge de 5 ans [**5**,**6**,**8**]. Les résultats de notre série rejoignent ceux de la littérature avec 80,88% des enfants de plus de 5 ans diagnostiqués pour ces tumeurs.

## Conclusion

Le profil épidémiologique et histopathologique des TCE dans la région de Marrakech au Sud Marocain se rapprochent assez sensiblement de ceux rapportés dans la littérature, particulièrement au Nord du pays et en Afrique du Nord. Toutefois, d'autres études multicentriques sont nécessaires dans notre pays pour mieux étayer et confirmer ces données. Cela permettra d'établir un registre national Marocain de TCE pouvant servir à d'autres études épidémiologiques à l'échelle continentale.

### Etat des connaissances actuelles sur le sujet

- Le profil épidémiologique et anatomopathologique des tumeurs cérébrales de l'enfant a été dressé à travers de multiples études internationales;
- De rares séries Marocaines ont aussi apporté des informations dans ce sens au Nord du pays.

### Contribution de notre étude à la connaissance

- Les particularités épidémiologiques et anatomopathologiques de ces tumeurs dans la région de Marrakech au Sud du Maroc sont rapportés ici pour la première fois.

## Conflits d’intérêts

Les auteurs ne déclarent aucun conflit d'intérêts.
